# RF Electromagnetic Field Treatment of Tetragonal Kesterite CZTSSe Light Absorbers

**DOI:** 10.1186/s11671-017-2183-9

**Published:** 2017-06-13

**Authors:** Mykola O. Semenenko, Ivan S. Babichuk, Oleksandr Kyriienko, Ivan V. Bodnar, Raquel Caballero, Maximo Leon

**Affiliations:** 1 V.Ye. Lashkaryov Institute of Semiconductor Physics, National Academy of Sciences of Ukraine, Prospect Nauky 41, 03680 Kyiv, Ukraine; 2 College of Optoelectronic Engineering, Key Laboratory of Optoelectronic Devices and Systems of Ministry of Education and Guangdong Province, Shenzhen University, 518060 Shenzhen, People’s Republic of China; 3 The Niels Bohr Institute, University of Copenhagen, Blegdamsvej 17, DK-2100 Copenhagen, Denmark; 4 Belarussky Gosudarstvenniy Universitet Informatiki i Radioelektroniki – BSU-BE, P. Brovki 6, 220013 Minsk, Belarus; 5 Photovoltaic Materials Group, Applied Physics Department, University Autonoma of Madrid, 28049 Madrid, Spain

**Keywords:** Tetragonal kesterite, Light absorber, Radio frequency treatment, Plasma etching, Kesterite FTIR investigation, Raman spectroscopy

## Abstract

In this work, we propose a method to improve electro-optical and structural parameters of light-absorbing kesterite materials. It relies on the application of weak power hydrogen plasma discharges using electromagnetic field of radio frequency range, which improves homogeneity of the samples. The method allows to reduce strain of light absorbers and is suitable for designing solar cells based on multilayered thin film structures. Structural characteristics of tetragonal kesterite Cu_2_ZnSn(S, Se)_4_ structures and their optical properties were studied by Raman, infrared, and reflectance spectroscopies. They revealed a reduction of the sample reflectivity after RF treatment and a modification of the energy band structure.

## Background

The problem of energy generation and accumulation becomes increasingly important both due to depletion of conventional sources of energy and increase of economical demands. This pushes forward the limits of alternative energy sources technology and, particularly, the technology of light-harvesting devices. Ranging from common Si-based solar cells (SCs) [1] to highly efficient although expensive III–V semiconductor-based SCs (single or multi-junction [2, 3]) and cheap but less efficient organic photovoltaic devices, the SC technologies remain in active search for optimal materials. At present, thin film SCs (TFSCs) based on kesterite structure Cu_2_ZnSn(S, Se)_4_ (CZTSSe) are being developed rapidly [4]. CZTSSe-based SCs have a number of advantages in contrast to other TFSCs (e.g., CuInGaSe_2_-based TFSC) being cost-efficient with respect to source components and non-toxic during the synthesis. The improved properties of Cu_2_ZnSnS_4_ (CZTS) include a direct band gap (about 1.5 eV) and a high absorption coefficient (above 10^4^ cm^−1^ in the visible spectral range), making it well suitable for photovoltaic applications [5]. Currently, the record efficiency of a prototype CZTSSe SC is 12.6% [6]. In order to increase the efficiency, several problems should be resolved. First, it is the non-stoichiometric composition of CZTSSe and the concentration of intrinsic defects. The second problem is a material degradation due to the coexistence of different crystallographic phases. Finally, it is the possible presence of the impurities of secondary binary and ternary compounds which are formed during the synthesis. Different phases present in material are hardly distinguishable mainly due to the imperfection of traditional methods of investigation [7]. These problems occur due to the small difference in cross-sections between the Cu and Zn in the X-ray scattering and similar diffraction patterns for kesterite, stannite, and their disordered phases. Therefore, it is difficult to determine the crystalline structure and the degree of structural disorder using X-ray diffraction (XRD) setup. Such information may be obtained by neutron diffraction [8] or synchrotron X-ray diffraction investigations [9]. As was demonstrated in Ref. [7], the power of beam using in XRD method cannot be fully exploited for the identification of secondary phases of ternary compounds in complex systems like CZTS. The same problem appears while distinguishing the structures of similar modifications with the same ternary or quaternary composition, e.g., kesterite and its “defect” modification or stannite. The intensity of the XRD reflex corresponds to the volume of a phase. Therefore, it is often impossible to distinguish tiny and typical broadening due to small size of the inclusion of the secondary phase peak when it is situated in the vicinity of the main peak of the principal phase. For this reason, researchers working in the field are looking for alternative but accessible methods for the identification and detection of the secondary phase. One of such promising methods is Raman spectroscopy. Application of such methods can simplify the post-processing methods for structural homogeneity improvement of CZTSSe materials. Moreover, analysis of structural properties represents an important technological task and is highly demanded for various photovoltaic applications. In Ref. [6], the high efficiency of SC was reached with record efficiency of 12.6% for CZTSSe. There, CZTSSe films were grown from the Sn and Cu chalcogenides dissolved in hydrazine solution as well as from the ZnS and ZnSe particles dispersed in the solution. Hydrazine was utilized to the growth process only, and post-growth treatment is performed by annealing in N_2_ and air, which allows dissolving certain precursors easily. However, it is highly toxic, and its explosive properties limit the potential usage. In this work, we propose a hydrazine-free method as a post-growth treatment for the improvement of the structural properties of light absorbers in the bulk and multilayered configurations. It is based on the application of hydrogen weak power plasma discharges using an electromagnetic field of radio frequency region.

## Methods

First, the method of radio frequency (RF) treatment was applied for silicon-based SCs in typical configuration. The area of diffusion field Si-SC was 2 cm^2^, and the layered structure consisted of (i) Al front grid, (ii) 50 nm thick anti-reflection Si_3_N_4_ layer, (iii) 30 nm thick charged dielectric SiO_2_ layer, (iv) inducted *n* ^++^ layer, (v) diffusion *n* ^+^ layer, (vi) quasi-neutral base area or *p*-Si, (vii) diffusion isotype junction or *p* ^+^ layer, and (viii) backside Al metallization. For measurements, the miniature SCs were collected in 10 groups. They were divided into three subgroups for future use as a reference, indoor and outdoor masks. During processing, samples were masked to avoid etching of the surface anti-reflection coatings. An inert gas was used as a mediator for RF beam. The SC samples were treated by 13.56 MHz RF beam. The initial sample (i.e., not subjected to the treatment) served as a reference. Variable parameters were the exposure time and the power of RF beam. The ranges of exposure time and beam power were 1–15 min and 0.19–2.25 W/cm^2^, respectively. The area of holder of RF reactor was 132 cm^2^. The hydrogen pressure in the chamber was fixed to 0.2 Torr. During depositions, the value of the voltage on the substrate was fixed (1900 V). Depositions were carried out at room temperature of holder. N_2_-based plasma treatment for pre-cleaning of the surfaces was performed using PlasmaEtch PE-50 XL (4.5′′W × 6′′D + 2.5′′ Clearance) with power of generator 150 W at 50 KHz.

Dark and illuminated (AM1.5) IU characteristics were measured using Kelvin probe with Keithley 2410h and LabTraser NI software assistance. To calculate the parameters of Si-SCs, we used the double-diode model following Ref. [10].

Next, RF treatment with optimal regimes was used in the processing of light-absorbing materials. RF-stimulated H^+^ plasma discharge with the source power of 0.8 W/cm^2^ was applied during 15 min. Sample surface was masked by Si wafer during the treatment. For our aims, we utilized three kinds of bulk CZTSSe with tetragonal structure. First, specimen type was obtained by the deposition of ZnS, CuS, and SnS binary compounds by flash evaporation on glass substrates with pre-deposited molybdenum as a bottom layer with subsequent annealing of the structure (see Ref. [11]). Samples of second type were grown by Bridgman method (vertical aligned zone) from respective source elements. In the next step, grown crystals were sputtered onto the glass substrates with and without molybdenum bottom layer by magnetron sputtering at different substrate temperatures and by electron beam evaporation (for SC manufacturing). The transmission/(n-R specular reflection) within the IR range was measured by FTIR spectrometer Infralum FT-801 in the 500–5000 cm^−1^ (0.06–0.5 eV) range: Specord-210 (A setup was configured as an attenuated total reflectance (ATR)), Shimadzu UV-3600 (B_s_ and B_d_ setups were configured as a specular/diffuse reflectance with integrating sphere of 100 mm), PerkinElmer Lambda-950 (C setup was configured as a diffuse reflectance with integrating sphere of 150 mm), UV-VIS-NIR Varian Cary 5000 (D setup was configured as a normal incidence beam for specular reflectance). A, B_s_, B_d_, C, and D configurations were used for UV, VIS, and NIR ranges, respectively. Absorption spectra were determined from the reflection spectra using dispersion integrals similar to well known method described in Refs. [11, 12]. To investigate the structural properties of the CZTSSe, *μ*-Raman spectroscopy (T64000 Horiba Jobin Yvon) was performed in backscattering configuration. For excitation of Raman spectra, the radiation of Ar^+^ laser with a wavelength of 514.5 nm was used. The power of laser irradiation was chosen sufficiently small (the power flux of the beam was 0.1 mW/μm^2^) to avoid change of the film structure during measurements. Raman spectra were recorded at room temperature, and the registration time was less than 1 min. Different parts of the sample were tested by several measurements for reproducibility and uniformity estimation. A ×50 objective of Olympus microscope was applied to focus onto the surface with diameter of spot less than 1 μm. Raman spectra were collected in different areas of each sample for accuracy, as non-uniform spots on the surface were visible under the light microscope. Collected results were averaged, and the nature of segregated crystalline phases was established.

## Results and Discussion

**Fig. 1:**
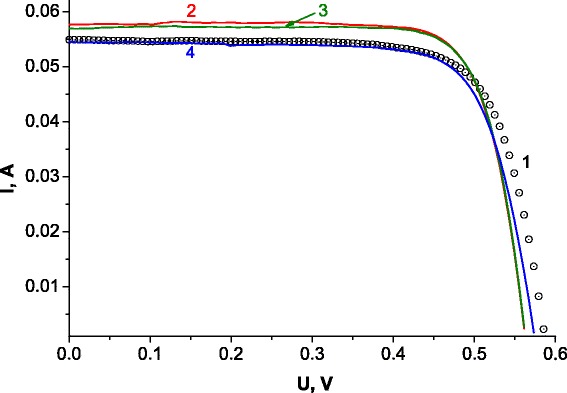
AM1.5 IU characteristics of Si-based SCs in general configuration under RF treatments (13.56 MHz stimulated discharge H^+^ plasma, *t* = 15 min, *P* = 0.8 W/cm^2^) with the following intensity values: *1* initial, *2* 95 W, *3* 225 W, *3* 225 W, and *4* 300 W

As a proof of a principle, we start to study the influence of RF for SCs treatment. The collected results are presented in Fig. 1.

Efficiency (*η*, %) and fill factor (FF) of Si-SCs were 11.692 and 0.746 (curve 1), respectively, and were improved after the treatments: 95 W = 12.337/0.775 (curve 2); 225 W = 12.291/0.783 (curve 3); 300 W = 11.458/0.752 (curve 4). Slopes of the curves 2 and 3 slightly differ from that corresponding to the initial sample (curve 1). We suppose this to be a result of degradation of Schottky contacts due to the heating occurring under RF. As can be seen from Fig. 1, the values of *U*_oc_ decreased but the values of *I*_sc_ increased. This can possibly happen due to the passivation of dangling bonds by highly reactive H atoms. Application of high-power RF treatment resulted in cracking of striped metallic contacts and destruction of p-n junction. This was observed in optical microscope, explaining the behaviour of curve 4 and its significant change. Thus, we assume that the proposed method can be applied for the modification of *η* and FF, but it should be optimized for TFSC improvement.

For sample characterization, we proceeded with the measurements of the reflectivity spectra. Generally, the absorption coefficient can be easily extracted from the measurements of transmission. However, there are difficulties in both accurate measurement of thickness and reflectivity losses in case of multilayered configuration of absorber, or if its appropriate thickness is less than 1 μm. For these reasons, it is highly desirable to make the second and independent method for measurement of absorption coefficient from measurements of reflectivity. Absorption coefficient is related to extinction coefficient by simple relation: *α*(*ω*, *E*) = 4*πk*(*ω*)*λ* ^− 1^ = 2*ωk*(*ω*)*c* ^− 1^ = 2*E*(*ℏc*)^− 1^ *k*(*E*), [*α*(*ω*, *E*)] = *cm* ^− 1^, where *k*(*ω,E*) is the extinction coefficient, *ω* is the angular frequency, *λ* is the wavelength, *c* is the speed of light, and *ℏ* is the reduced Planck’s constant, respectively. The complex reflection amplitude can be written using Fresnel equations, and in the case of normal incidence reads

1 $$ r=\frac{n_0-\left({n}_1+ ik\right)}{n_0+\left({n}_1+ ik\right)}, $$

where *n*_0_ is the refraction index of media for an incident beam (*n*_0_ ≥ 1), and material refraction is characterized by the complex refractive index *n* = *n*_1_ + *ik*. While *r* is a complex reflectivity and is not measured itself, it can be easily decomposed as any complex number using Euler’s formula:

2 $$ \begin{array}{l} r=\sqrt{R}{e}^{i\theta}; rr*=\sqrt{R}{e}^{i\theta}\sqrt{R}{e}^{- i\theta}=\frac{n_0-\left({n}_1+ ik\right)}{n_0+\left({n}_1+ ik\right)}\frac{n_0-\left({n}_1- i k\right)}{n_0+\left({n}_1- i k\right)}=1-\frac{4{n}_0{n}_1}{{\left({n}_0+{n}_1\right)}^2+{k}^2}= R;\\ {} R={\left| r\right|}^2={\left(\sqrt{A^2+{B}^2}\right)}^2; \tan \left(\delta \right)=\frac{B}{A}=\frac{2{n}_0 k}{n_1^2+{k}^2-{n}_0^2},\begin{array}{c}\hfill \delta =\left(\theta -\pi \right),\kern1em \left({n}_0 k\ge 1\right);\hfill \\ {}\hfill \delta =\left(\theta +\pi \right),\kern1em \left({n}_0 k<1\right),\hfill \end{array}\\ {}\end{array} $$

where *R* is the ratio of the intensities of reflected and incident light beams that can be measured directly, *θ* is the phase of reflected light, *A* and *B* are the real and imaginary components of complex reflectivity, and *n*_1_ and *k* are the refraction and extinction indices of absorber, respectively.

Eq. (1) can be rewritten by direct decomposition into real and imaginary parts as

3 $$ r=\frac{{n_0}^2-{n_1}^2-{k}^2}{{\left({n}_0+{n}_1\right)}^2+{k}^2}+ i\frac{\left(-2{n}_0 k\right)}{{\left({n}_0+{n}_1\right)}^2+{k}^2}= A+ i B. $$

If we know *R* and *θ* are transformed by the algorithm used in Refs. [11, 12], the solution of the system of Eq. (2) gives

4 $$ \begin{array}{l}{n}_1=\frac{K^2 N+4{n_0}^2 N\mp K\sqrt{\left({K}^2+4{n_0}^2\right)\left({N}^2-4{n_0}^2\right)}}{2\left({K}^2+{N}^2\right)},\\ {} k=\frac{K^2 N-4{n_0}^2 K\mp N\sqrt{\left({K}^2+4{n_0}^2\right)\left({N}^2-4{n_0}^2\right)}}{2\left({K}^2+{N}^2\right)},\end{array} $$

where auxiliary coefficients are

$$ N=\frac{4{n}_0}{1- R}-2{n}_0, K=\frac{2{n}_0}{ \tan \theta}. $$

In the region where the oscillator strengths for the optical transitions are mostly exhausted the dielectric function can be represented by the classical Drude formula [13, 14]:

5 $$ \varepsilon \left(\omega \right)={\varepsilon}_{\infty }-\frac{{\omega_p}^2}{\omega \left(\omega + i\gamma \right)};\sigma \left(\omega \right)=\frac{{\omega_p}^2}{\gamma + i\omega};{\omega}_p=\sqrt{\frac{q^2{N}_{\nu}}{\varepsilon_0{m}^{*}}};\mu \left(\omega \right)=\frac{\sigma \left(\omega \right)}{N_{\nu} q};\gamma =\frac{1}{\tau}, $$

6 $$ {\sigma}_r\left(\omega \right)={\varepsilon}_0\omega {\varepsilon}_{im}\left(\omega \right);{\sigma}_{im}\left(\omega \right)={\varepsilon}_0\omega \left({\varepsilon}_{\infty }-{\varepsilon}_r\left(\omega \right)\right), $$

where *σ*(*ω*) is the complex optical conductivity (lowercase indices *r* and *im* denote real and imaginary part, respectively), *ω*_*p*_ is the plasma frequency of the valence electrons, m* is the free electron mass, *N*_v_ is the effective density of the valence electrons, *τ* is the average collision time, and *ε*_0_ is the vacuum permittivity, respectively. All these parameters should be attributed to the value of plasma frequency using the sum rule: $\frac{1}{2}\pi {\omega_p}^2={\displaystyle \underset{0}{\overset{\infty }{\int }}\frac{\omega {\varepsilon}_{im}}{\varepsilon_r^2+{\varepsilon}_{im}^2} d\omega}.$

**Fig. 2:**
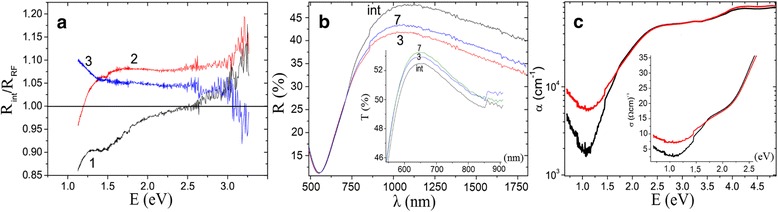
Optical spectra of CZTSSe before and after RF treatments. **a** *1* Ratio of reflectances for bulk CZTS processed from metallic precursors (A setup); *2* ratio of reflectances for glass/Mo/Cu/CZTSe (A setup); *3* reflectance of bulk CZTS processed from sulfide precursors (B_d_ setup). **b** Reflectance and transmittance (*insert*) of CZTS with respect to plasma exposure (C setup) with the steps of 1, 3, and 7. **c** Spectra of absorbance of CZTS thin films with (*black*) and without (*red*) RF treatment during 3 min (C setup). *Insert*: optical conductivity spectra of the same films

Transformed optical spectra of R(E)_initial_/R(E)_RF_ of CZTSSe corresponding to different technological conditions are shown in Fig. 2a. Analysis showed that the reflection of structures after RF treatment decreased in the frequency range of 1.2 to 3 eV in the case of multilayered structure (curves 2 and 3) and in the range of 2.4 to 3.3 eV (curve 1) for bulk structures. The mismatch of the improvement ranges occurs due to post-processed free sample for bulk (curve 1) and the presence of Schottky contacts or hetero-junctions for layered sample (curves 2 and 3). It shall be noted that transformation of spectra following the procedure of Ref. [15] would not be correct without correction terms depending on the measurement configuration of beams. In the case of A setup, ATR setup changes of the period of complex phase angle influences on the determination of the complex refractive index and should be corrected. Using non-ATR technique, the actual phase shift *θ*_act_ can be obtained similar to the procedure described in Ref. [15]. In our experiments, the best prediction of refractive index was realized to D setup, slightly worse to B_s_ setup, and difficult to A setup. This depends on the transitions during multi-reflections from mirrors. It was found to be impossible doing adequate estimation of phase angle in C setup due to the diffuse integrating sphere. Thus, we conclude that the determination of absorption coefficient as well as pseudo-optical functions from the reflectivity measurements is correct for the measurement of normal incidence single-beam absolute specular reflection. Otherwise, all results must be attributed to the parameters obtained by direct method (e.g., Brewster angle-based technique).

The next stage of experiments included transmission and reflection measurements of the films on the glass with lateral dimensions larger than typical aperture of the beams of double-beam spectrophotometers. For this aim, the bulk CZTS was evaporated by electron beam and then additionally treated by RF plasma. The step for exposition was 1 min. Respective reflectance and transmittance (insert) spectra are illustrated in Fig. 2b, in accordance with exposure ratio. The maximal effect has been revealed for the sample exposure time of 3 min (curve 3).

After that, the corresponding absorption coefficients and ratios between the initial optical conductivities were calculated by Eqs. (3) and (6) using the result obtained by the most effective method. They are illustrated in Fig. 2c and in the inset in this figure, respectively.

A least-squares estimation of nonlinear parameters can be done by minimizing procedure using the following relations:

7 $$ \left\{\begin{array}{l} T=\frac{{\left(1- R\right)}^2}{e^{\alpha d}-{R}^2{e}^{-\alpha d}};\\ {} R=1-\frac{4{n}_0{n}_1}{{\left({n}_0+{n}_1\right)}^2+{k}^2};\\ {} k=\frac{\alpha \left(\lambda, E\right)}{4\pi \lambda}.\end{array}\right. $$

Here, the first relation is known as Beer’s law in the case of multi-reflections in parallel plate and the second one is the square of the absolute value of complex reflectivity.

As can be seen from Fig. 2c, the light-absorbing properties of CZTS increased after RF treatment mainly within the band gap. The value of optical conductivity can be evaluated using the assumption in the Drude model of conductivity as well as the plasma frequency parameter corresponding to the treatments. In the case of RF treatment, its value is 2.294 eV which is slightly higher than that for initial case (2.278 eV). Based on these results, we assume that RF treatment improves the absorption. But the presence of Cu-rich and other metal-enriched components results in poor electronic properties, and treatment condition should be optimized by additional cleaning.

**Fig. 3:**
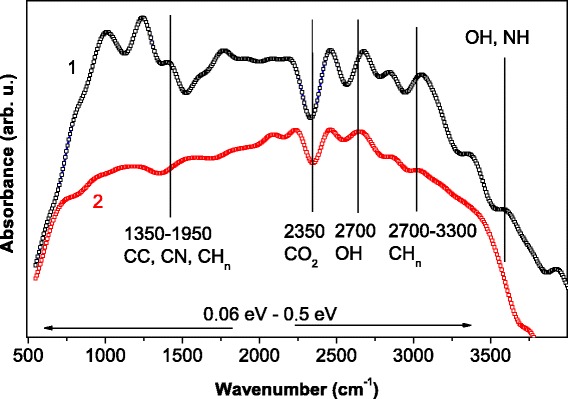
FTIR spectra of bulk CZTS sample with (curve 1) and without (curve 2) RF treatment (13.56 MHz stimulated discharge H^+^ plasma, *t* = 15 min, *P* = 0.8 W/cm^2^)

To estimate the role of plasma components during the treatment, FTIR technique was applied. Absorption spectra are presented in Fig. 3. Absorption bands for bulk CZTS_4_ with and without RF treatment ranged from 500 to 4000 cm^−1^ (wave numbers). These bands include C–N (1250 cm^−1^, 1600 cm^−1^); *sp* ^2^ hybridized bonds (1490–1650 cm^−1^) of C–C, C=C stretching bands; stretching band of CH_*n*_ at 2870 and 3100 cm^−1^, corresponding to *sp* ^*n*^ hybridized bonds; CO_2_ (2350 cm^−1^); and 2700 and 3600 cm^−1^ attributed to water and organic components [16]. As we can see, RF treatment resulted in the reduction of absorption in the whole spectral range. In the case of absorption by *sp* ^2^ hybridized bonds for C–C and C=C units at 1500–1650 cm^−1^, the explanation is well known. Normally, graphite-like phases being exposed to H^+^ plasma are removed from the structures [16]. The decrease of intensity for absorption band related to symmetric oscillations of CH_3_ bond (at 2872 cm^−1^), CH, and CH_2_ (2900–2926 cm^−1^) can be explained by the reduction of hydrogen concentration in the film. Thus, H^+^ ions remove the components of impurities due to its high mobility even if the sample is masked without accumulation of *sp* ^*n*^ hybridized compositions.

**Fig. 4:**
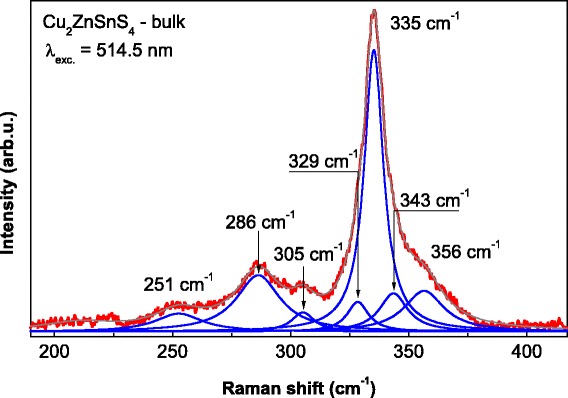
Raman spectrum of bulk CZTS sample with Lorentzian fits (*I* ~ 0.5 mW; *λ* = 514.5 nm)

The Raman spectra of the bulk CZTS were deconvoluted on Lorentzian components and are presented in Fig. 4. The two dominant peaks at 286 and 335 cm^−1^ and the bands at 251, 305, 343, and 356 cm^−1^ were attributed to A, E, and B symmetry modes, respectively. Their positions were similar to those in the experimental results described in Refs. [17–19], and their symmetry assignment was consistent with theoretical calculations reported in Refs. [20, 21]. Fitting the Raman spectrum by a set of components, we can assume that a weaker component around 329 cm^−1^ is observed at the low-frequency side for the most intense band (335 cm^−1^). This Raman band can be assigned to the disordering of Zn and Cu atoms in CZTS lattice as was discussed in Ref. [22]. This disordering is often caused by so-called anti-site defects such as Zn atoms replacing Cu (Cu_Zn_) and vice versa (Zn_Cu_). The influence of phase on the change of Raman spectra for kesterite is discussed in Ref. [22]. The disordering degree for kesterite structure can be estimated using intensity ratio I_329_/I_335_ of the peaks at 329 and 335 cm^−1^. In our case, this ratio was 0.11 and is comparable to the values obtained for thin films described in [22]. It should be noted that the Raman spectra changes for light and dark areas are negligibly small that correlates with Ref. [23].

**Fig. 5:**
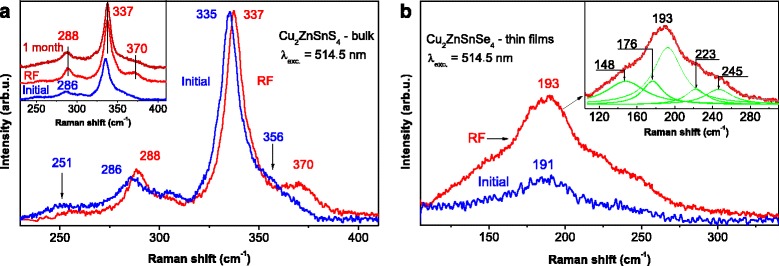
Raman spectra of bulk samples before (*blue curves*) and after (*red curves*) RF treatment for materials. **a** CZTS (*inset* shows the spectra before, straight after, and 1 month after RF treatment). **b** CZTSe film deposited onto Cu/Mo coated glass (*inset* shows the deconvolution by Lorentzian fits)

Raman spectra of CZTS and Cu_2_ZnSnSe_4_ (CZTSe) samples after RF treatments are shown in Fig. 5a, b respectively. They are marked blue and red corresponding to initial and RF treated samples, respectively. As can be seen from Fig. 5a (red line), the position of the band at 286 cm^−1^ is shifted to the high-frequency region by 2 cm^−1^, and its half-width is decreased by almost two times (22 cm^−1^), resulting in the increase of intensity of the band. In Ref. [24], Suragg et al. suggested a hypothesis that the I_286_/I_305_ ratio may be used for the determination of the ordering of compound. Uniform compound is characterized by the higher ratio value and vice versa. Applying this assumption, the intensity of the band increase of ratio I_288_/I_305_ and its correlation with our results (the decrease of the ratio I_331_/I_337_) was established. Both values indicate the structure ordering of the compound. As can be seen, the most intense band at 335 cm^−1^ for A symmetry shifts by 2 cm^−1^ after the treatment, but its half-width remains equal to 10 cm^−1^ corresponding to that of untreated sample. We assume that all improvements appeared due to the ordering of kesterite crystal lattice. The disordered kesterite has a structure like the stannite and manifests in the spectrum as a band at 331 cm^−1^ [23]. Our assumption is based on the decrease of the ratio I_331_/I_337_ equal to 0.06 [22]. In the inset in Fig. 5a, we demonstrate three curves and show that the RF-inducted changes are stable in time anyways within 1-month period as indicated by the stability of the main band positions. At the same time, the band at 370 cm^−1^ is corresponding to CZTS and being visible after the treatment disappeared during this period. The increase of the band intensity at 370 cm^−1^ with respect to that of the initial samples was associated with the RF treatment, since after 1-month storage in air the band intensity has decreased.

Similar treatment was provided to CZTSe processed in multilayered configuration, and its deconvoluted spectra are shown in Fig. 5b. The spectrum is characterized by the presence of two main peaks at 193 and 176 cm^−1^ identified as the main resonances in CZTSe [25] as well as weaker CZTSe specific peaks located at 223 and 245 cm^−1^. The frequency band of 223 cm^−1^ corresponds to the oscillation of E symmetry kesterite-like structure of CZTSe, a band with a frequency of 245 cm^−1^ that corresponds to B symmetry of kesterite-like structure [21, 26]. Unlike CZTSSe, there are no distinct spectral features that can be associated with technological conditions. Secondary phase positions mainly for ZnSe and Cu_2_SnSe_3_ differ from those discussed in Ref. [25–27], in our case without any significant second phases. The electron beam evaporation of bulk samples in this case was performed on a substrate under the heating up to 190 °C [28] without additional annealing to reach stoichiometry. The conditions depended on the use of organic substrate during subsequent processing. Nevertheless, RF treatment also resulted in the positive effect for the spectrum of CZTSe whose main band was shifted by 2 cm^−1^ from 191 cm^−1^ (blue curve) to 193 cm^−1^ (red curve). This gives reasons to assume that influence of the treatment has a similar effect for both materials and is associated with the partial reduction of structural defects.

## Conclusions

In this work, we applied hydrogen-based weak power plasma discharges using radio frequency (13.56 MHz) electromagnetic field treatment for the improvement of the optical properties of bulk and thin film kesterite samples. The structural characteristics and optical properties were studied by Raman, FTIR, and normal incidence reflection spectroscopy. It was shown that the position of main kesterite band (286 and 335 cm^−1^ for CZTS) shifted to the high-frequency region by 2 cm^−1^ and its full-width at half maximum decreased by almost two times (for the 286 cm^−1^ mode). This results in the increase of the band intensity. Similar shift by 2 cm^−1^ with respect to the main band of A symmetry appeared in the Raman scattering of CZTSe thin films. The analysis showed that the improvements resulted from the ordering of the crystal lattice and were stable during 1-month period. FTIR spectroscopy showed that sample treatments removed carbon-based impurities and inhibited accumulation of *sp* ^*n*^ hybridized compositions. Reflection spectra were transformed into absorption spectra using the dispersion integrals in the visible spectral range. This allowed estimating pseudo-optical function, Drude conductivity, and carrier mobility change, as well as concentration before and after plasma treatments. Therefore, plasma treatment resulted in not only surface cleaning from organic inclusions but also relieved internal stress. Such processing can be performed inside vacuum chambers during the post-processing stage. We conclude therefore that proposed hydrazine-free method of treatment can be applied for the creation of light absorbers with reduced strain and is suitable for the production of thin film multilayered solar cell.

## Abbreviations

CZTS: Cu_2_ZnSnS_4_
CZTSe: Cu_2_ZnSnSe_4_
CZTSSe: Cu_2_ZnSn(S, Se)_4_
FTIR: Fourier transform infrared spectroscopy
IR: Infrared
RF: Radio frequency
SCs: Solar cells
TFSCs: Thin film solar cells
XRD: X-ray diffraction

## References

[1] Saga T (2010). Advances in crystalline silicon solar cell technology for industrial mass production. NPG Asia Materials.

[2] Yablonovitch E, Miller OD, Kurtz SR (2012) The opto-electronic physics that broke the efficiency limit in solar cells. Photovoltaic Specialists Conference (PVSC), IEEE 38th:001556; doi:10.1109/PVSC.2012.6317891

[3] King RR, Law DC, Edmondson KM, Fetzer CM, Kinsey GS, Yoon H, Sherif RA, Karam NH (2007). 40% efficient metamorphic GaInP/GaInAs/Ge multijunction solar cells. Appl Phys Lett.

[4] Abermann S (2013). Non-vacuum processed next generation thin film photovoltaics: towards marketable efficiency and production of CZTS based solar cells. Solar Energy.

[5] Friedlmeier TM, Wieser N, Walter T, Dittrich H, Schock HW (1997) Heterojunctins based on Cu_2_ZnSnS_4_ and Cu_2_ZnSnSe_4_ thin films. Proceedings of the 14-th European Photovoltaic Specialists Conference. Barcelona. 1242–1245

[6] Wang W, Winkler MT, Gunawan O, Gokmen T, Todorov TK, Zhu Y, Mitzi DB (2014). Device characteristics of CZTSSe thin-film solar cells with 12.6% efficiency. Adv Energy Mater.

[7] Walsh A, Chen S, Wei S-H, Gong X-G (2012). Kesterite thin-film solar cells: advances in materials modelling of Cu_2_ZnSnS_4_. Adv Energy Mater.

[8] Schorr S, Gonzalez G (2009). In-situ investigation of the structural phase transition in kesterite. Phys Stat Sol A.

[9] Schorr S, Hoebler H-J, Tovar M (2007). A neutron diffraction study of the stannite-kesterite solid solution series. Eur J Mineral.

[10] Ishaque K, Salam Z, Taheri H (2011). Simple fast and accurate two-diode model for photovoltaic modules. Solar Energy Materials and Solar Cell.

[11] Babichuk IS, Yukhymchuk VO, Semenenko MO, Klyui NI, Caballero R, Hreshchuk OM, Lemishko IS, Babichuk IV, Ganus VO, Leon M (2014). Optical and morphological properties of tetragonal Cu_2_ZnSnS_4_ thin films grown from sulphide precursors at lower temperatures. Semiconductor Physics, Quantum Electronics & Optoelectronics.

[12] Yamamoto K, Masui A, Ishida H (1994). Kramers-Kronig analysis of infrared reflection spectra with perpendicular polarization. Appl Optics.

[13] Pitarke JM, Silkin VM, Chulkov EV, Echenique PM (2007). Theory of surface plasmons and surface-plasmon polaritons. Rep Prog Phys.

[14] Kyriienko O, Shelykh IA (2011). Angle-resolved reflectance and surface plasmonics of the MAX phases. Opt Lett.

[15] Bertie JE, Lan Z (1996). An accurate modified Kramers–Kronig transformation from reflectance to phase shift on attenuated total reflection. J Chem Phys.

[16] Semenenko M, Okrepka G, Yilmazoglu O, Hartnagel HL, Pavlidis D (2010). Electrical conditioning of diamond-like carbon films for the formation of coated field emission cathodes. Appl Surf Sci.

[17] Fontane X, Izquierdo-Rosa V, Saucedo E, Schorr S, Yukhymchuk VO, Valakh MY, Perez-Rodriguez A, Morante JR (2012). Vibrational properties of stannite and kesterite type compounds: Raman scattering analysis of Cu_2_(Fe,Zn)SnS_4_. Journal Alloys and Compounds.

[18] Dumcenco D, Huang YS (2013). The vibrational properties study of kesterite Cu_2_ZnSnS_4_ single crystals by using polarization dependent Raman spectroscopy. Opt Mater.

[19] Dimitrievska M, Fairbrother A, Izquerdo-Roca V, Suacdeo E, Perez-Rodrigez A, Fontane X, Jawhari T (2014). Multiwavelength excitation Raman scattering study of polycrystalline kesterite Cu_2_ZnSnS_4_ thin films. Appl Phys Lett.

[20] Himmrich M, Haeuseler H (1991). Far infrared studies on stannite and wurtzstannite type compounds. Spectrochimica Acta.

[21] Gürel T, Sevik C, Cagin T (2011). Characterization of vibrational and mechanical properties of quaternary compounds Cu_2_ZnSnS_4_ and Cu_2_ZnSnSe_4_ in kesterite and stannite structures. Phys Rev B.

[22] Caballero R, Garcia-Llamas E, Merino JM, León M, Babichuk I, Dzhagan V, Strelchuk V, Valakh M (2014). Non- stoichiometry effect and disorder in Cu_2_ZnSnS_4_ thin films obtained by flash evaporation: Raman scattering investigation. Acta Mater.

[23] Valakh MYa, Kolomys OF, Ponomaryov SS, Yukhymchuk VO, Babichuk IS, Izquierdo-Rosa V, Saucedo E, Perez-Rodriguez A, Morante JR, Schorr S, Bodnar IV (2013) Raman scattering and disorder effect in Cu_2_ZnSnS_4_. Physica Status Solidi RRL 7(4):258-261

[24] Scragg JJS, Choubrac L, Lafond A, Ericson T, Platzer-Björkman C (2014). A low-temperature order-disorder transition in Cu_2_ZnSnS_4_ thin films. Appl Phys Lett.

[25] Fairbrother A, Fontané X, Izquierdo-Roca V, Placidi M, Sylla D, Espindola-Rodriguez M, López-Mariño S, Pulgarín FA, Vigil-Galán O, Pérez-Rodríguez A, Saucedo E (2014). Secondary phase formation in Zn-rich Cu_2_ZnSnSe_4_-based solar cells annealed in low pressure and temperature conditions. Prog Photovolt: Res Appl.

[26] Yao L, Ao J, Jeng M-J, Bi J, Gao S, He Q, Zhou Z, Sun G, Sun Y, Chang L-B, Chen J-W (2014). CZTSe solar cells prepared by electrodeposition of Cu/Sn/Zn stack layer followed by selenization at low Se pressure. Nanoscale Res Lett.

[27] Dzhagan V, Litvinchuk A, Kruszynska M, Kolny-Olesiak J, Valakh M, Zahn D (2014). Raman scattering study of Cu_3_SnS_4_ colloidal nanocrystals. J Phys Chem C.

[28] Park D, Nam D, Jung S, An S, Gwak J, Yoon K, Yun JH, Cheong H (2011). Optical characterization of Cu_2_ZnSnSe_4_ grown by thermal co-evaporation. Thin Solid Films.

